# Supplementary material for: The role of veterinary diagnostic laboratories during COVID-19 response in the United States
Source: PLoS One. 2024 Jun 25;19(6):e0303019. doi: 10.1371/journal.pone.0303019 (PMC11198799; doi:10.1371/journal.pone.0303019)
Supplement: S1 File — Following formal survey approval by the American Association of Veterinary Medical Colleges, this online cross-sectional Qualtrics survey was distributed to American Association of Veterinary Medical Colleges and the National Animal Health Laboratory Network member laboratories in the United States. (DOCX) [file pone.0303019.s001.docx]

**S1 File. Survey Distributed to Veterinary Diagnostic Laboratories.**

As researchers at the Cornell University College of Veterinary Medicine and Cornell’s Department of Public and Ecosystem Health, we are gathering information about the involvement of United States veterinary diagnostic laboratories during the COVID-19 pandemic response. You are being asked to participate in a research study titled, ***“Exploring the Role of Veterinary Diagnostic Laboratories in the United States During COVID-19.”*** Your response to this survey is voluntary and will take about 20 minutes to complete. **We ask that you please complete this survey by April 14, 2023.**More information is provided below.
**Purpose of research**
This study is designed to describe the role of veterinary diagnostic laboratories in the United States during the COVID-19 pandemic. This survey is not anonymous because we hope to collect specific information about relevant veterinary diagnostic laboratories. We intend to publish these results and will share the preprint of the paper with all participants.
**What this survey involves**
If you decide to participate, you will be asked if your institution conducted animal and human SARS-CoV-2 testing and, if so, some details about the tests your institution performed.
**Risks and benefits**
There are no appreciable risks from participating in this study. The information that is gathered from this study may benefit future emergency responses to zoonotic infectious agents by describing the contributions of veterinary diagnostic laboratories during the COVID-19 pandemic response.
**Participant rights**
Participation in this study is voluntary and you may decide not to participate or to discontinue your participation at any time. A decision not to participate or withdraw will not affect your current or future relationship with Cornell University. As informed by Cornell University’s Institutional Review Board (IRB), because this research is focused on institutional methods and responses, it is not considered human participation research and is therefore exempt from IRB review.
**If you have questions**
The lead researcher conducting this study is Dr. Lorin Warnick Dean of Veterinary Medicine and Professor in the Department of Population Medicine and Diagnostic Sciences at Cornell University. If you have questions, you may contact him at ldw3@cornell.edu or 607-253-3771. You can also direct questions to Nia Clements at nmc72@cornell.edu, Master of Public Health candidate at Cornell and co-investigator.

***If you agree to participate, responding to the survey questions constitutes your consent.***

1. What is your name? *[free response]*
2. What is your position title? *[free response]*
3. What was/is your role in SARS-CoV-2 testing at your institution? *[free response]*
4. What is the name of your institution? *[free response]*
5. Please briefly describe the organizational structure of your animal health diagnostic laboratory (e.g. state funded institution separate from a university or college, unit within a college of veterinary medicine, other structure). *[free response]*
6. To whom does your diagnostic laboratory director report? *[free response]*
7. Is your laboratory part of the National Animal Health Laboratory Network?

Yes

No

Unsure

1. Did your institution conduct **animal** SARS-CoV-2 testing?

Yes

No

1. Did your institution conduct **human** SARS-CoV-2 testing (either diagnostic or screening tests)?

Yes

No

Display This Question:

If Did your institution conduct animal SARS-CoV-2 testing? = No

And Did your institution conduct human SARS-CoV-2 testing (either diagnostic or screening tests)? = No

*“Thank you for answering these initial questions. There are no further questions. Your response is complete.”*

Display This Question:

If Did your institution conduct animal SARS-CoV-2 testing? = Yes

*“The next series of questions are displayed because you answered that your institution conducted* ***animal*** *SARS-CoV-2 tests.”*

1. **For animal SARS-CoV-2 testing,** what species did your institution test? *[free response]*
2. **For animal SARS-CoV-2 testing,** when did your institution *begin* testing? *[free response]*
3. **For animal SARS-CoV-2 testing,** when did your institution *end* testing? Please enter "ongoing" if testing is still being offered. *[free response]*
4. **For animal SARS-CoV-2 testing,** how many *total* animals did your institution test, from the beginning of testing through March 31, 2023? *[free response]*
5. **For animal SARS-CoV-2 testing,** what type of SARS-CoV-2 tests did your institution perform?

RT-PCR

Other (Explain)

1. **For animal SARS-CoV-2 testing,** did your laboratory perform viral nucleic acid sequencing to gather genotypic information about the viral variants?

Yes

No

Other (Explain)

Display This Question:

If Did your institution conduct human SARS-CoV-2 testing (either diagnostic or screening tests)? = Yes

*“The next series of questions are displayed because you answered that your institution conducted* ***human*** *SARS-CoV-2 diagnostic and/or screening tests.”*

1. **For human SARS-CoV-2 testing,** who was testing conducted for? Select all that apply.

University population

General community

Other (Explain)

1. **For human SARS-CoV-2 testing,** did your institution *require* testing for the university community?

Yes

No

Other (Explain)

Not applicable

Display This Question:

If For human SARS-CoV-2 testing, did your institution require testing for the university community? = Yes

1. Please briefly describe this testing requirement. *[free response]*

-------------------------------------------------------------------------------------------------------------------------------

1. **For human SARS-CoV-2 testing,** please describe the reasons that your laboratory performed testing (e.g., providing university or regional COVID-19 testing capacity, tracking variants, research purposes only). *[free response]*
2. **For human SARS-CoV-2 testing,** what type of SARS-CoV-2 tests did your institution perform?

RT-PCR

Other (Explain)

Display This Question:

If For human SARS-CoV-2 testing, what type of SARS-CoV-2 tests did your institution perform? = RT-PCR

1. If RT-PCR was used, which target genes were used in the RT-PCR test? *[free response]*

-------------------------------------------------------------------------------------------------------------------------------

1. **For human SARS-CoV-2 testing,** which sample types were tested? Select all that apply.

Saliva

Nasopharyngeal swab

Anterior nares swab

Other (Explain)

1. **For human SARS-CoV-2 testing,** was your laboratory responsible for sample collection?

Yes

No

Other (Explain)

Display This Question:

If For human SARS-CoV-2 testing, was your laboratory responsible for sample collection? = Yes

1. Please describe how sample collection was performed (e.g. self-collection, observed testing stations, etc). *[free response]*

-------------------------------------------------------------------------------------------------------------------------------

1. **For human SARS-CoV-2 testing,** were samples pooled for testing?

Yes

No

Other (Explain)

Display This Question:

If For human SARS-CoV-2 testing, were samples pooled for testing? = Yes

1. Please describe your institution’s pooling methodology. *[free response]*

-------------------------------------------------------------------------------------------------------------------------------

1. **For human SARS-CoV-2 testing,** was automation or robotics used to achieve and/or increase testing capacity?

Yes

No

Other (Explain)

Display This Question:

If For human SARS-CoV-2 testing, was automation or robotics used to achieve and/or increase testing... = Yes

1. Please describe your institution’s automation or robotics methodology. *[free response]*

-------------------------------------------------------------------------------------------------------------------------------

1. **For human SARS-CoV-2 testing,** did your laboratory perform viral nucleic acid sequencing to gather genotypic information about the viral variants?

Yes

No

Other (Explain)

1. **For human SARS-CoV-2 testing,** when did your institution *begin* testing? *[free response]*
2. **For human SARS-CoV-2 testing,** when did your institution *end* testing? Please enter “ongoing” if testing is still being performed.  *[free response]*
3. **For human SARS-CoV-2 testing,** how many people could be tested *per day* at highest capacity? *[free response]*
4. **For human SARS-CoV-2 testing,** how many *total* samples did your institution test from the beginning of testing to March 31, 2023? *[free response]*
5. **For human SARS-CoV-2 testing,** how long did it typically take to return test results? *[free response]*
6. **For human SARS-CoV-2 testing,** how many days per week was your laboratory open and what was your laboratories operational hours? *[free response]*
7. **For human SARS-CoV-2 testing,** under what regulatory framework did your laboratory provide testing? Select all that apply.

Collaborated with a human facility with Clinical Laboratory Improvement Amendments (CLIA) certification

Independently obtained CLIA certification

Performed testing under FDA emergency authorization for COVID-19 screening

1. Which of the following were barriers in planning and executing **human** SARS-CoV-2 testing? Select all that apply.

Obtaining the proper certifications and/or licensure

Achieving satisfactory test accuracy

Limited human workforce capacity

Limited testing equipment, materials and supplies

Limited physical space for testing

Obtaining funding

Other organizational responsibilities

Difficulty coordinating with other organizations

Other (Explain)

1. Are you willing to be contacted to further discuss your institution's human SARS-CoV-2 testing during COVID-19 response?

Yes

No

Display This Question:

If Are you willing to be contacted to further discuss your institution's human SARS-CoV-2 testing du... = Yes

1. Please provide your contact information. *[free response]*

-------------------------------------------------------------------------------------------------------------------------------

1. If you have any additional information you would like to share, including links to news articles, websites or scientific publications describing your testing program, please enter it here. *[free response]*
