# Supplementary material for: The role of veterinary diagnostic laboratories during COVID-19 response in the United States
Source: PLoS One. 2024 Jun 25;19(6):e0303019. doi: 10.1371/journal.pone.0303019 (PMC11198799; doi:10.1371/journal.pone.0303019)
Supplement: S2 File — To learn more about the specific elements of a laboratory’s response, a convenience sample of survey respondents were asked to participate in a semi-structured interview. (DOCX) [file pone.0303019.s002.docx]

**S2 File. Interview Questions.**

**Interview questions for group 1: laboratories that conducted human SARS-CoV-2 testing**

1. Optimizing testing capacity: Could you explain the methods you used to achieve testing capacity?
   1. [If applicable] Why were robotics or automation not used?
   2. [If applicable] Why did you decide against pooling?
2. Barriers to laboratory operation: Can you expand more on what barriers were the most difficult to overcome for you all?
   1. [If applicable] Did you have an existing relationship with the human health facility you collaborated with?
3. What information would be useful for you, moving forward, with this publication?
4. [If time permits]: what would you do differently in the future, if another pandemic arose? In other words, what were the major learning outcomes from this experience?

**Interview questions for group 2: laboratories that did not conduct human SARS-CoV-2 testing**

1. What are the regular operations or scope of your laboratory?
2. [If applicable] Was there a COVID-19 testing program on campus? If so, who did the testing?
   1. [Follow-up] Was there ever any consideration of using the veterinary laboratory for this testing response?
3. Optimizing testing capacity: Could you explain the methods you used to achieve testing capacity on a regular basis?
4. Barriers to laboratory operation: What barriers would you imagine there would be in setting up a COVID-19 lab, if you were asked to participate in the COVID-19 response?
5. What information would be useful for you, moving forward, with this publication?
6. [If time permits]: what would you do differently in the future, if another pandemic arose? In other words, what were the major learning outcomes from this experience?
